# Theoretical Investigation of a Novel Two-Dimensional Non-MXene Mo_3_C_2_ as a Prospective Anode Material for Li- and Na-Ion Batteries

**DOI:** 10.3390/ma17153819

**Published:** 2024-08-02

**Authors:** Bo Xue, Qingfeng Zeng, Shuyin Yu, Kehe Su

**Affiliations:** 1 School of Physical Science and Technology, Northwestern Polytechnical University, Xi’an 710129, China; 2 MSEA International Institute for Materials Genome, Langfang 065500, China; zengqf@dianyunkeji.com (Q.Z.); yusy@dianyunkeji.com (S.Y.); 3 Particle Cloud Biotechnology (Hangzhou) Co., Ltd., Hangzhou 310018, China; 4 Science and Technology on Thermostructural Composite Materials Laboratory, Northwestern Polytechnical University, Xi’an 710072, China; 5 School of Chemistry and Chemical Engineering, Northwestern Polytechnical University, Xi’an 710129, China

**Keywords:** anode material, structure prediction, transition metal carbide, energy capacity

## Abstract

A new two-dimensional (2D) non-MXene transition metal carbide, Mo_3_C_2_, was found using the USPEX code. Comprehensive first-principles calculations show that the Mo_3_C_2_ monolayer exhibits thermal, dynamic, and mechanical stability, which can ensure excellent durability in practical applications. The optimized structures of Li*_x_*@(3×3)-Mo_3_C_2_ (*x* = 1–36) and Na*_x_*@(3×3)-Mo_3_C_2_ (*x* = 1–32) were identified as prospective anode materials. The metallic Mo_3_C_2_ sheet exhibits low diffusion barriers of 0.190 eV for Li and 0.118 eV for Na and low average open circuit voltages of 0.31–0.55 V for Li and 0.18–0.48 V for Na. When adsorbing two layers of adatoms, the theoretical energy capacities are 344 and 306 mA h g^−1^ for Li and Na, respectively, which are comparable to that of commercial graphite. Moreover, the Mo_3_C_2_ substrate can maintain structural integrity during the lithiation or sodiation process at high temperature. Considering these features, our proposed Mo_3_C_2_ slab is a potential candidate as an anode material for future Li- and Na-ion batteries.

## 1. Introduction

With the advancement of nanoengineering and microelectronics technology, the exploration of energy-storage devices based on two-dimensional (2D) materials has driven a tremendous amount of research [1]. It is well known that the performance of rechargeable batteries is strongly influenced by the properties of their electrodes. Due to the unique features of 2D materials, such as the large specific surface area, high mechanical strength, high charge carrier capability, and good flexibility [2], rechargeable batteries using 2D materials as electrodes are regarded as a highly promising technology that can effectively integrate clean energy (e.g., solar, wind, and tidal energy) with electricity grids [3]. The ongoing transition from fossil fuels to renewable energy is the key to lowering the CO_2_ footprint [4]. Currently, the most widely used secondary batteries are lithium-ion batteries (LIBs). Because the shortage of lithium resources is an obstacle to developing LIBs and because sodium is more Earth-abundant and much cheaper than lithium, sodium-ion batteries (SIBs) are a potentially viable battery technology to supplement LIBs [5,6,7].

Among all possible candidates for high-performance electrode materials, MXenes, a large family of 2D transition metal carbides (TMCs) and nitrides (TMNs), are promising candidates for advanced energy-storage devices, such as LIBs, lithium–sulfur batteries, and supercapacitors [1,8,9]. The general formula of MXenes is M*_n_*_+1_X*_n_*T*_x_* (*n* = 1–3), where M donates the transition metal (e.g., V, Mo, Ti, Nb, or Cr), X is nitrogen and/or carbon, and T*_x_* presents the surface termination (e.g., -O, -F, or -OH) [10]. Usually, MXenes can be synthesized by selectively etching the A layer from MAX phases (A represents the group IIIA/IVA element) [10]. Many MXenes have been identified as excellent anodes for secondary batteries, with low diffusion barriers, low operating voltage, and high storage capacity [1,11,12,13]. For example, as an electrode material for LIBs, surface halogenated Ti_3_C_2_ MXenes were investigated using first-principles calculations [14]. The results indicated that the Ti_3_C_2_T_2_ MXenes exhibit metallic conductivity with high structural stability and mechanical strength. Compared with Ti_3_C_2_F_2_ and Ti_3_C_2_Br_2_, Ti_3_C_2_Cl_2_ exhibits a large elastic modulus, a low diffusion barrier (0.275 eV), a high open circuit voltage (0.54 eV), and an energy capacity (674.21 mA·h g^−1^), which is beneficial for high performance as an electrode. Li et al. [15] prepared a Co-doped MXene (Co@MXene) anode for LIBs and the anode exhibits an excellent reversible capacity of 1283.2 mA h g^−1^ at a current density of 0.1 A g^−1^ after 120 cycles. Apart from LIBs, a great research effort has also been devoted to the application of MXenes in SIBs [16,17,18]. For example, Fan et al. [19] theoretically studied the properties of the V_3_C_2_ MXene as an anode for SIBs and found that the diffusion energy barrier for Na is 0.02 eV, indicating a high rate for charge/discharge processes. The theoretical Na capacity of V_3_C_2_ is 606.42 mA h g^−1^, suggesting a great potential in SIBs. Hence, these research findings indicate that the MXenes possess high potential value in LIBs and SIBs.

It is important to further search for TMC/TMN monolayers that can be used as high-performance electrode materials for rechargeable batteries. Thanks to the considerable progress in the computational algorithms and the computational power of modern computers, the theoretical prediction based on first-principles calculations offers an efficient way to explore energetically stable 2D materials as new electrodes. Xu et al. [20] predicted a new stable 2D TMC containing C_2_ dimers, VC_2_, using the swarm-intelligent global-structure search method. As an anode material for LIBs, the VC_2_ sheet shows a high Li-storage capacity of 1073 mA h g^−1^ for multilayer adsorption, while stacked VC_2_ possesses an even larger capacity of 1430 mA h g^−1^. At the same time, the Li atoms stored in the interlayer of VC_2_ can migrate easily with a low barrier of 0.09 eV. Using the swarm structural search method, Yu et al. [21] extensively explored Ta-C monolayers with various Ta*_x_*C*_y_* compositions (*x* = 1 and *y* = 1–4, or *x* = 2 and *y* = 1). They determined the stable, carbon-rich TaC_2_ as a promising anode for LIBs. The theoretical capacity of the metallic TaC_2_ reaches 523 mA h g^−1^ when adsorbing two layers of Li atoms. In addition, the resultant performance of the diffusion barrier and working voltage are better than that of commercial graphite. Wu et al. [22] designed two TMC/TMN monolayers as anodes for LIBs, namely tetr-V_2_C_2_ and tetr-V_2_N_2_. They reported Li storage capacities of 851 and 824 mA h g^−1^ for tetr-V_2_C_2_ and tetr-V_2_N_2_, respectively. These two structures also exhibit low diffusion barriers for Li atoms (smaller than 0.1 eV). Generally, it is very important for the further improvement of rechargeable batteries to predict new types of 2D TMCs/TMNs as potential electrodes.

To our knowledge, so far, only the M_O3_C_2_ MXene has been reported [23], with a Mo/C ratio of 3:2. It is, therefore, meaningful to explore new stable structures possessing the same elemental ratio as the Mo_3_C_2_ MXene. In this work, a new stable 2D molybdenum carbide, Mo_3_C_2_, was predicted using the crystal structure search technique associated with the first-principles calculations. Although the 2D structure has the same chemical formula as the Mo_3_C_2_ MXene, its configuration is completely different from the previously reported MXene. The performance of the predicted Mo_3_C_2_ as an anode for LIBs/SIBs was comprehensively scrutinized through theoretical calculations.

## 2. Computational Details

Based on the evolutionary algorithm, the structural prediction of the 2D Mo_3_C_2_ was performed with the USPEX code, which allows researchers to efficiently find possible stable structures according to a given chemical composition [24,25]. Specifically, in order to ensure structural diversity in the search process using the USPEX, a large Mo/C elemental ratio of 6:4 was chosen to search low-energy structures, and 60 structures were randomly created in the first generation. The population size of every ensuing generation was set to 60, in which the fractions of structures produced by the heredity, softmutation, transmutation, and random structure generator were 60, 20, 10, and 10%, respectively. The structure relaxation and energy calculation were based on the density functional theory (DFT), as implemented in the VASP 5.4 package [26,27]. The generalized gradient approximation (GGA) [28] in the form of the Perdew–Burke–Ernzerhof (PBE) [29] exchange correction functional was employed to describe the exchange–correlation interactions. For the good structures found with the USPEX, the refined structures and properties were calculated with the VASP package. An energy cutoff of 500 eV was adopted to expand wave functions into plane waves, and the computations were converged within 10^−5^ eV and 0.02 eV/Å for energy and force criteria, respectively. A vacuum distance of 15 Å (z direction) was used to prevent interaction between adjacent monolayers. The van der Waals interaction was taken into account using the DFT-D3 method [30]. A 3 × 3 supercell was selected to calculate the adsorption and diffusion of Li/Na atoms on the Mo_3_C_2_ monolayer. The Brillouin zone was sampled with 10 × 10 × 1 and 5 × 5 × 1 *k*-point meshes for the unit cell and supercell, respectively. Using the Bader charge method [31], the partial charges of different atoms were calculated, and the amount of charge transfer was estimated quantitatively. The climbing image nudged elastic band (CI-NEB) [32] was employed to investigate the potential migration pathways and energy barriers of adatoms on the substrate.

To examine the dynamical stability of the Mo_3_C_2_ monolayer, the phonon frequency was analyzed using the Phonopy code [33]. In addition, ab initio molecular dynamics (AIMD) simulations with the Andersen thermostat [34] and the *NVT* ensemble were carried out to verify the thermal stability of the studied structures. The size of the supercell of the Mo_3_C_2_ was 4 × 5 for the phonon–dispersion relationship calculation and AIMD simulations.

## 3. Results and Discussion

### 3.1. Structure and Stability

**Figure 1:**
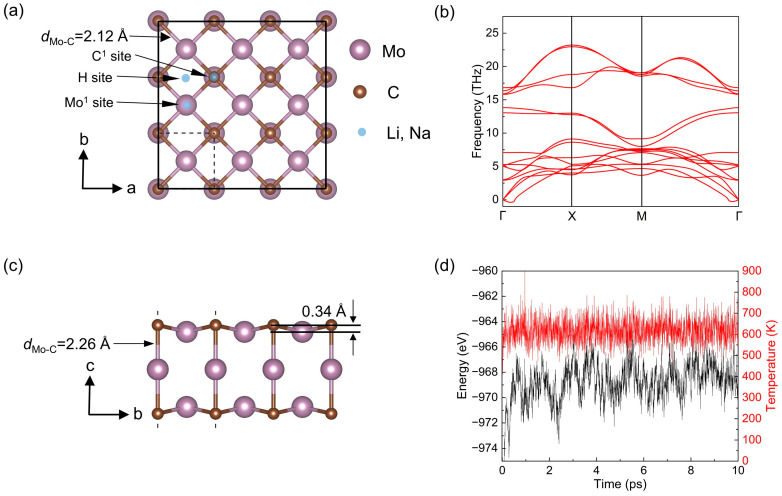
(**a**) Top and (**c**) side views of the 2D p4m Mo_3_C_2_ monolayer, where C^1^ (top of C), H (hollow), and Mo^1^ (top of Mo) represent three possible adsorption sites for adatoms, and the dashed and solid lines represent the unit cell and the 3 × 3 supercell, respectively. (**b**) Phonon dispersion spectra of the Mo_3_C_2_. (**d**) Energy and temperature as a function of time for Mo_3_C_2_ during the AIMD simulations.

After the search with the USPEX, a square p4m structure, Mo_3_C_2_, was found to be the most energetically stable configuration. Figure 1a,c present the geometric configuration of Mo_3_C_2_ associated with the three adsorption sites considered for the Li and Na atoms. The optimized Mo_3_C_2_ has the lattice parameters of *a* = *b* = 2.954 Å, and the thickness of the monolayer is 4.523 Å. The in-plane Mo-C bond length is 2.117 Å, smaller than the interatomic distance of 2.261 Å in the z direction. It should be noted that the surface of the monolayer is not absolutely planar, with a buckled height of 0.344 Å for each surface. Different from the configuration of the Mo_3_C_2_ MXene [23], where every sub-layer contains only one type of element, the surface sub-layers in our predicted Mo_3_C_2_ contain both molybdenum and carbon species. It should be noted that, although the Mo atoms in the middle layer of the Mo_3_C_2_ slab are only two-fold coordinated with their neighboring C atoms, the large gaps surrounding the Mo atoms can accommodate the electrons of the Mo atoms, resulting in a suitable environment for them. All the C atoms are five-fold coordinated with their neighboring Mo atoms. Similar five-fold coordination can also be found in other stable 2D transition metal carbides, such as TaC [21] and tetr-V_2_C_2_ [22]. Notably, all the C atoms are exposed on the surfaces, which can facilitate the adsorption of the Li/Na atoms [35].

Cohesive energy *E*_coh_ refers to the energy required to separate solids into free atoms and can be used to evaluate the bond strength of the structure [36,37]. *E*_coh_ can be calculated as [22]

(1) $$E_{\mathrm{coh}}=[E(A_{m}B_{n})-mE(A)-nE(B)]/(m+n)$$

where *E*(A*_m_*B*_n_*), *E*(A), and *E*(B) represent the energies per formula unit of A*_m_*B*_n_* and isolated atoms A and B, respectively. In general, a smaller *E*_coh_ value reflects better stability for the corresponding structure. Here, the calculated *E*_coh_ for the predicted Mo_3_C_2_ is −8.45 eV/atom, higher than that of the Mo_3_C_2_ MXene (−8.28 eV/atom), suggesting its high energetic stability. For the dynamical stabilities, the phonon spectra of the Mo_3_C_2_ have no obvious imaginary mode in the Brillouin zone (Figure 1b), indicating that our predicted Mo_3_C_2_ is dynamically stable. In addition, AIMD simulations were used to examine the thermal stability of the predicted Mo_3_C_2_ at 600 K with a time step of 3 fs. As illustrated in Figure 1d, the energies and temperatures of the system fluctuated around the equilibrium positions. At the end of the 10 ps simulations, the framework of the Mo_3_C_2_ maintained its initial configuration well (Figure S1), demonstrating its high thermal stability at high temperatures.

A promising electrode material should have sufficient in-plane rigidity to resist deformation. The four elastic constants (*C*_11_, *C*_22_, *C*_12_, and *C*_66_) [38] and orientation-dependent Young’s moduli (*Y*) of the predicted Mo_3_C_2_ are listed in Table 1. The in-plane 2D Young’s moduli in the Cartesian [01] and [10] directions are [38]

(2) $$Y_{\left[01\right]}^{2D}=\frac{C_{11}C_{22}-C_{12}^{2}}{C_{11}}\;\mathrm{and}\;Y_{\left[10\right]}^{2D}=\frac{C_{11}C_{22}-C_{12}^{2}}{C_{22}}$$

The elastic constants and *Y* of the 2D Mo_3_C_2_ MXene, graphene [38], BN [38], and SiC [38] are included for comparison. All the elastic constants of the predicted Mo_3_C_2_ meet the Born–Huang elastic stability criteria, *C*_11_*C*_22_ − *C*_12_^2^ > 0 and *C*_66_ > 0 [39], indicating mechanical stability. Compared with other monolayers, the *Y* values of our predicted Mo_3_C_2_ are much larger than those of the Mo_3_C_2_ MXene, BN, and SiC, and slightly higher than those of graphene. All these results suggest that the predicted Mo_3_C_2_ is robust enough to form a free-standing membrane.

### 3.2. Lithiation and Sodiation Processes: Adsorption and Electronic Property

**Figure 2:**
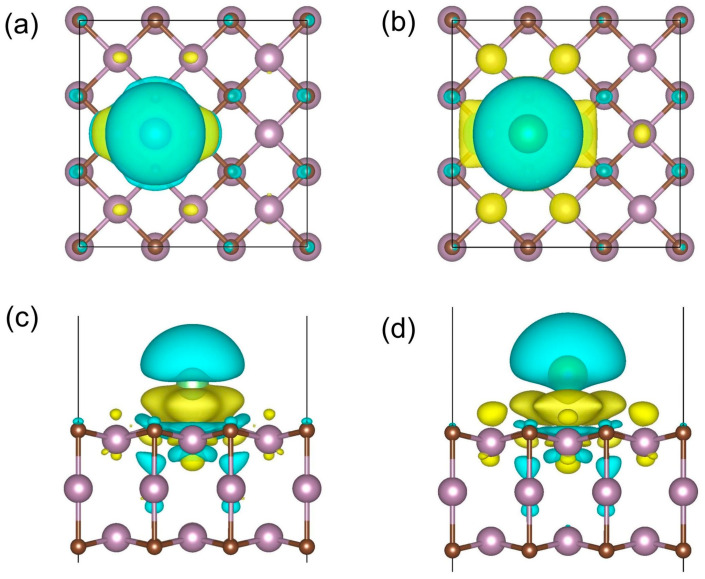
Differential charge density distributions for the H site (hollow 4-fold site): (**a**,**c**) Li@(3×3)-Mo_3_C_2_; (**b**,**d**) Na@(3×3)-Mo_3_C_2_ plotted with isovalue = 8 × 10^−4^ electrons/Bohr^3^. Yellow denotes charge accumulation, and blue represents depletion.

**Figure 3:**
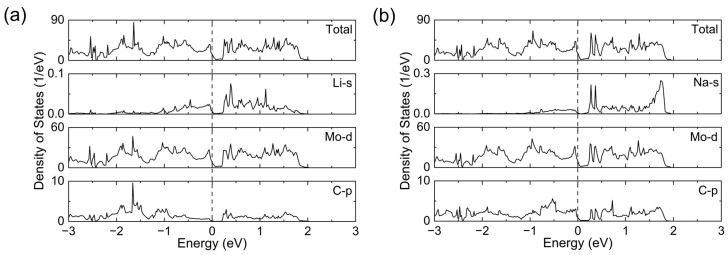
Total and partial densities of states of (**a**) Li@(3×3)-Mo_3_C_2_ and (**b**) Na@(3×3)-Mo_3_C_2_ with the adatom at the H site. The Fermi level is set to zero and marked with the dashed line.

In this work, a 3 × 3 supercell was adopted to study the lithiation and sodiation of the predicted 2D Mo_3_C_2_. According to the symmetry of the Mo_3_C_2_ monolayer, three representative adsorption sites for M (Li or Na), namely C^1^ (on-top C), H (4-fold hollow), and Mo^1^ (on-top Mo), were considered to explore the Li/Na-adsorption properties (Figure 1a). The adsorption energy *E*_ad_ of M was estimated via the following expression [19]:

(3) $$E_{\mathrm{ad}}=(E_{M_{x}@(3\times {3)-\mathrm{Mo}}_{3}C_{2}}-E_{(3\times {3)-\mathrm{Mo}}_{3}C_{2}}-xE_{M})/x$$

where $E_{M_{x}@(3\times {3)-\mathrm{Mo}}_{3}C_{2}}$ (M = Li or Na), $E_{(3\times {3)-\mathrm{Mo}}_{3}C_{2}}$, and $E_{M}$ are the energies of the Li/Na-adsorbed (3×3)-Mo_3_C_2_ sheet, the pristine (3×3)-Mo_3_C_2_ substrate, and a Li/Na atom in the bulk structure, respectively. In addition, the variable *x* is the number of adsorbed alkali atoms. For a single adatom, the adsorption energies for all the considered sites are listed in Table 2, and the optimized configurations with the lowest *E*_ad_ are shown in Figure 2a and Figure 3a. For both Li- and Na-adsorbed systems, the H site is the most favorable adsorption site, as it has the smallest *E*_ad_ of −0.646 and −0.617 eV for Li and Na, respectively. On the other hand, the Mo^1^ site has the maximum *E*_ad_ for all the systems, indicating the weakest adsorption strength. Apart from adsorption energy, the adatom height (*h*) can also reflect the adsorption strength for different sites. The values of *h* for single-adatom adsorption on different sites are listed in Table 2. For C^1^ and Mo sites, *h* is the vertical distance between the adatom and the surface C or Mo atom directly below the adatom. In contrast, for the H site, *h* is the distance between the adatom and the center of two surface C atoms around the H site. The H site has the smallest *h* of 1.66 Å for Li and 2.14 Å for Na, suggesting the strongest adsorption strength. The height at Mo^1^ is significantly larger than that at C^1^ and H sites, with *h* values of 2.47 and 2.76 Å for Li and Na, respectively. These results indicate that the adsorption strength for a single adatom is highest at site H, followed by the C^1^ site and then the Mo^1^ site. This strength order is consistent with the corresponding order obtained from the adsorption energy.

As the charges of surface atoms can influence adsorption site preference, Bader charges for the pristine Mo_3_C_2_ and single-adatom adsorbed systems were calculated and are listed in Table 3. In the pristine Mo_3_C_2_, the C atoms (C^1^ sites) are negatively charged, leading to an affinity with the electropositive Li/Na atoms. Conversely, the Mo atoms (Mo^1^ sites) are positively charged, causing weak adsorption for adatoms. In addition, the electron density depletion of 4-fold hollow sites (H sites) allows the localization of alkali metal atoms. To visualize the charge distribution in single-adatom adsorption systems, the differential charge density, ∆*ρ*, was calculated, and the results are shown in Figure 2. The electrons tend to transfer from the adatoms to the substrate, introducing a built-in electric field to the adsorbed systems. The number of electrons transferred from Li to the substrate is 0.1 e larger than from Na to the substrate (Table 3).

As the electronic structure can influence the battery performance of the anode material, we analyzed the density of states (DOSs) of the Mo_3_C_2_ and single-adatom adsorbed Mo_3_C_2_, with the adatom located on the stable H site. As shown in Figure S2, a high DOS peak near the Fermi level can be observed for the predicted Mo_3_C_2_ monolayer, implying its excellent electronic conductivity. The partial DOSs curves indicate that the DOS at the Fermi level is mainly contributed by the Mo-d and C-p orbitals. There is an obvious overlap near the Fermi level between the Mo-d and C-p orbitals, indicating strong p-d orbital hybridization between the C and Mo species. After adsorbing a metal atom, the adsorbed systems also exhibit metal characteristics (Figure 3). The DOS at the Fermi level is dominated by the Mo-d orbital in the Li@(3×3)-Mo_3_C_2_, while in the Na@(3×3)-Mo_3_C_2_ it is dominated by the Mo-d and C-p orbitals. It is noteworthy that the systems maintain a metallic nature after adsorption, which can ensure good electronic conduction and is indispensable for an ideal battery electrode.

**Figure 4:**
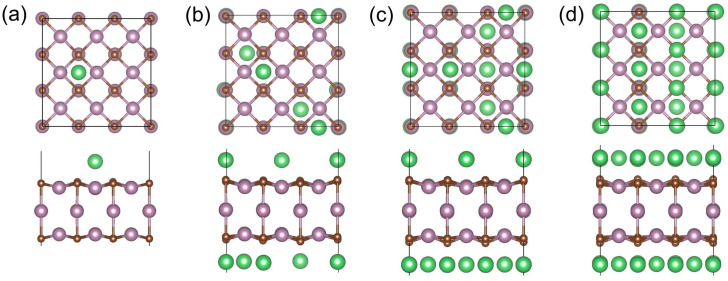
Top and side views of the 3×3 supercells for the most stable configuration of Li*_x_*@(3×3)-Mo_3_C_2_ at *x* = (**a**) 1, (**b**) 6, (**c**) 12, and (**d**) 18.

**Figure 5:**
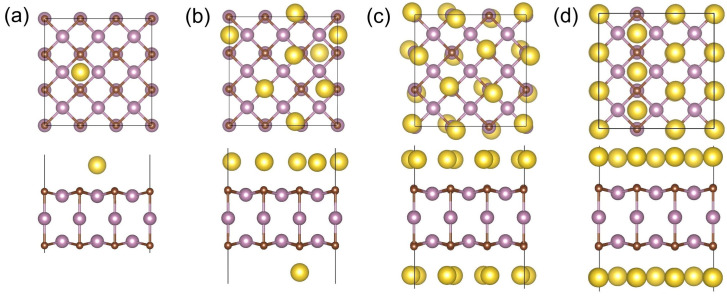
Top and side views of the 3 × 3 supercells for the most stable configuration of Na*_x_*@(3×3)-Mo_3_C_2_ at *x* = (**a**) 1, (**b**) 6, (**c**) 12, and (**d**) 18.

Next, we investigated the configurations of adsorbing multiple Li/Na atoms. Figure 4 shows the most stable configurations of the Li*_x_*@(3×3)-Mo_3_C_2_ at *x* = 1, 6, 12, and 18, and Figure S3 displays the optimal structures at *x* = 1–18. As the number of adsorbed Li atoms increases, the number of adatoms located on the top of the C atoms (C^1^ sites) rises gradually, and the C^1^ sites become the majority at *x* > 9. In addition, the Li atoms cannot be stabilized on top of the Mo atoms. For the saturated concentration of the first layer on the (3×3)-Mo_3_C_2_, the structure with the lowest energy contains 6 Li atoms on the H sites and 12 Li atoms on the C^1^ sites. For the Na-adsorbed systems, Figure 5 shows the most stable configurations of the Na*_x_*@(3×3)-Mo_3_C_2_ at *x* = 1, 6, 12, and 18, and Figure S5 presents every optimized structure at *x* = 1–18. Similarly to the Li-adsorbed systems, the most common adsorption site types for Na are C^1^ and H, and the Na_18_@(3×3)-Mo_3_C_2_ contains 6 Na atoms on the H sites and 12 Na atoms on the C^1^ sites. It is noteworthy that the adsorption energy of the optimal configurations for the saturated concentration is 0.023 eV for Li and 0.010 eV for Na, lower than the configurations where all the adatoms are adsorbed on the C^1^ sites. As shown in Figure 4b and Figure 5b, for *x* = 6, multiple Li atoms can be found on both sides of the substrate, while the Na atoms are mostly adsorbed on one surface. This is related to the fact that the substrate and previous adatoms have a joint impact on the adsorption positions of subsequent adatoms. On the one hand, the substrate and previous adatoms have an adsorption effect for the following adatoms. On the other hand, the accumulation of charge transferred from the alkali atoms on the surface reduces the adsorption effect. Different from the first adatom, the second Li/Na atom tends to be adsorbed on another surface due to weak attraction from the first adatom. Due to the Bader charges of different atoms, as shown in Table 3, Li transfers more charge to the substrate than Na atoms. Compared with the Na counterpart, in the Li-adsorbed system, a greater charge accumulation on the surface of the substrate will result in a weaker attraction for subsequent adatoms. This results in the adsorption of multiple Li atoms on both sides of the Mo_3_C_2_ slab (Figure S3). For the Na-adsorbed system, when the number of adatoms ranges from 4 to 7, the adsorption effect from previous adatoms slightly decreases due to a small amount of charge accumulation, resulting in most of the Na atoms being adsorbed on the same surface (Figure S5). When the number of Na adatoms is greater than 7, the charge accumulation is big enough to result in adsorption on both surfaces. For one-layer adsorption, the lattice constants increase from 8.834 Å for the (3×3)-Mo_3_C_2_ to 8.886 Å (about 0.59% tensile strain) for the Li_18_@(3×3)-Mo_3_C_2_ and to 8.924 Å (about 1.02% tensile strain) for the Na_18_@(3×3)-Mo_3_C_2_. The small expansions in the Li/Na adsorption processes suggest excellent substrate stability.

The adsorption of the second layer of Li/Na atoms on the Mo_3_C_2_ substrate with the first layer of adatoms was also studied, and the corresponding configurations are displayed in Figures S4 and S6 for Li and Na, respectively. The results show that the Li atoms tend to be located on the hollow sites or near the top of the Mo atoms. A maximum of 18 Li atoms are deposited on the Li_18_@(3×3)-Mo_3_C_2_, resulting in two Li monolayers per substrate surface. With regard to the Na atoms on the Na_18_@(3×3)-Mo_3_C_2_, the majority tend to migrate to the positions near Mo^1^ sites. The maximum number of Na atoms in the second layer is 14, less than that of Li atoms in the Li-adsorbed system. This difference is due to the larger radius of sodium compared to lithium. For double-layer adsorption, the lattice constants are 8.864 Å for the Li_36_@(3×3)-Mo_3_C_2_ and 8.928 Å for the Na_32_@(3×3)-Mo_3_C_2_. It is worth noting that the lattice constant for the double-layer adsorption of lithium atoms is smaller than for the single-layer adsorption of lithium atoms. This may be due to the strong interaction between the Li atoms. Generally, the adsorption of Li/Na atoms has little effect on the lattice expansion of the substrate, which is beneficial for improving the life span of the electrode.

### 3.3. Li/Na Surface Diffusion

**Figure 6:**
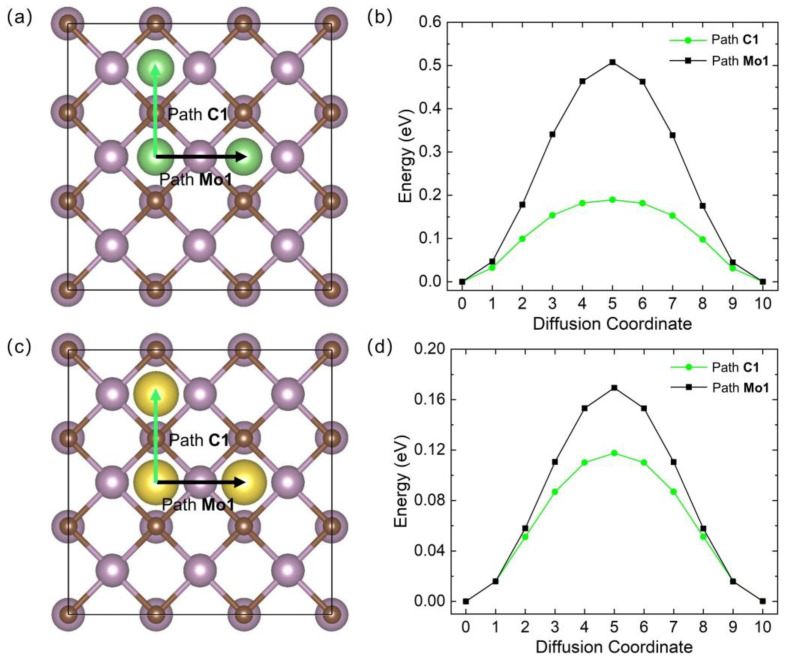
The migration pathways of (**a**) Li and (**c**) Na on the Mo_3_C_2_ monolayer. The energy profiles of (**b**) Li and (**d**) Na diffusion on the Mo_3_C_2_ monolayer.

It is believed that the diffusion barriers of adatoms on the surface of the substrate influence the charge/discharge rate of the electrode. In this work, as shown in Figure 6a,c, two diffusion pathways between two adjacent H sites were considered and labeled path **C1** (through the H-C^1^-H line) and path **Mo1** (through the H-Mo^1^-H line). The CI-NEB [31] method was applied, and nine images were adopted to calculate the migration energy barriers. For the Li-adsorbed system, the **C1** pathway has the lowest energy barrier of 0.190 eV (Figure 6b), which is much smaller than for path **Mo1** (0.508 eV). In the case of Na diffusion, the adatom tends to migrate through path **C1** with a barrier of 0.118 eV, which is similar to Li diffusion. The activation barrier for path **Mo1** (0.169 eV) is approximately three times smaller than for Li. Compared with the Li atom, the larger distance between the Na atom and the surface of the Mo_3_C_2_ sheet can effectively reduce the diffusion barriers for Na. Therefore, the diffusion barriers for Li are higher than for Na overall. The lowest barriers for Li/Na on the Mo_3_C_2_ are remarkably smaller than those on typical 2D materials, such as graphene (0.31 eV for Li [40] and 0.18 eV for Na [41]) and silicene (0.28 eV for Li [42] and 0.25 eV for Na [43]). These low energy barriers are highly beneficial to applying the predicted Mo_3_C_2_ in LIBs and SIBs.

### 3.4. Theoretical Storage Capacity, Open-Circuit Voltage, and Thermal Stability

The specific energy capacity of anode material is proportional to the number of adsorbed adatoms. The storage capacity of the predicted 2D Mo_3_C_2_ host with two layers of adatoms was investigated in this work. The maximum theoretical capacity (*C*_M_) can be obtained using the following formula [19]:

(4) $$C_{M}=zw_{\max}F/M_{{\mathrm{Mo}}_{3}C_{2}}$$

where *z* is the number of valence electrons of the adatom, *w*_max_ is the maximum adatom concentration, *F* is Faraday’s constant (26,801 mA h mol^−1^), and $M_{{\mathrm{Mo}}_{3}C_{2}}$ is the molar mass of the Mo_3_C_2_. After adsorbing two layers of adatoms, the Mo_3_C_2_ can adsorb up to 36 Li atoms and 32 Na atoms, corresponding to the concentrations of 4 and 3.56 for Li and Na, respectively. The maximum theoretical storage capacities of the Mo_3_C_2_ were calculated as 344 mA h g^−1^ for Li and 306 mA h g^−1^ for Na, which are comparable to that of commercial graphite (372 mA h g^−1^) [44]. The results suggest that the 2D Mo_3_C_2_ can serve as an anode material for LIBs and SIBs.

The charge/discharge processes of the Mo_3_C_2_ sheet can be described by the following half-cell reaction [19]:

Mo_3_C_2_ + *w*M^+^ + *w*e^−^ ↔ Mo_3_C_2_M*_w_* (5)

The open-circuit voltage (OCV) is estimated via the following equation [45]:

(6) $$\mathrm{OCV}\approx (E_{{\mathrm{Mo}}_{3}C_{2}}+wE_{M}-E_{{\mathrm{Mo}}_{3}C_{2}M_{w}})/wze$$

**Figure 7:**
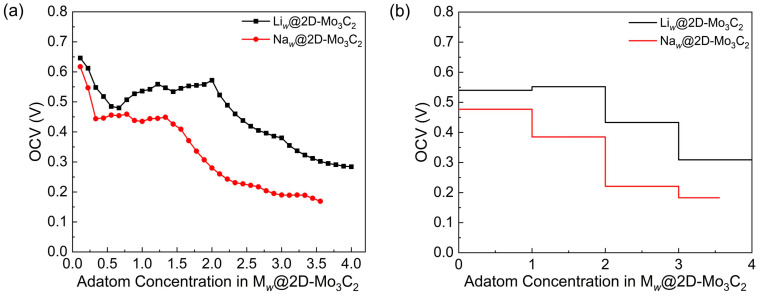
(**a**) Open-circuit voltage and (**b**) average open-circuit voltage as a function of the concentration of adatoms in M*_w_*@2D-Mo_3_C_2_.

The calculated OCV values, as a function of the concentration of adatoms on the Mo_3_C_2_ supercell, are presented in Figure 7a. The highest voltages for Li and Na are 0.65 and 0.62 V, respectively, corresponding to the single adatom on the Mo_3_C_2_ supercell. For both Li- and Na-adsorption systems, the OCV curves decrease initially and tend to be relatively moderate in the following stage. Subsequently, the voltage curves once again display a downward trend from concentrations *w* = 2 for Li and *w* = 1.33 for Na. In addition, Figure 7b displays the average OCV values of M*_w_*@2D-Mo_3_C_2_ for four regions of the entire concentration range. The average OCV values for all the systems generally decline as the concentration of adatoms increases, with ranges of 0.31 to 0.55 V and of 0.18 to 0.48 V for Li*_w_*@2D-Mo_3_C_2_ and Na*_w_*@2D-Mo_3_C_2_, respectively. Generally, it is accepted that the OCV values for anode materials are between 0.1 and 1.0 V [22]. Thus, the OCV results suggest that the 2D Mo_3_C_2_ is a potential candidate as an anode material for LIBs and SIBs.

**Figure 8:**
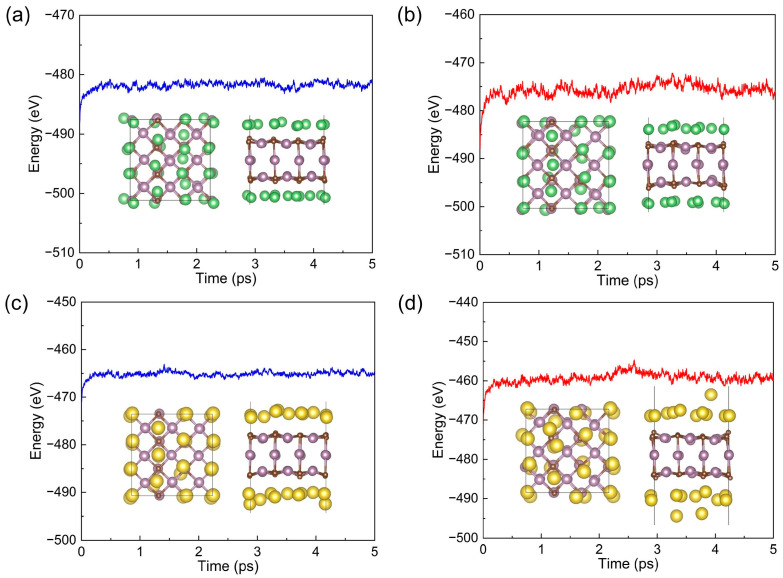
Energies as a function of time of (**a**) Li_18_@(3×3)-Mo_3_C_2_ at 300 K, (**b**) Li_18_@(3×3)-Mo_3_C_2_ at 600 K, (**c**) Na_18_@(3×3)-Mo_3_C_2_ at 300 K, and (**d**) Na_18_@(3×3)-Mo_3_C_2_ at 600 K during the AIMD simulations (inset: the structures after 5 ps AIMD simulations).

AIMD simulations for the Li_18_@(3×3)-Mo_3_C_2_ and Na_18_@(3×3)-Mo_3_C_2_ at temperatures of 300 and 600 K were carried out with a time step of 3 fs to study the reliability of the anode material under different temperatures. The energy fluctuations and final configurations are presented in Figure 8. The energies of the systems do not display big fluctuations around the equilibrium position. For AIMD simulations at 300 K, the structures exhibit no significant deformation. In the case of 600 K, although there is a significant change in the position of adsorbed metal atoms, the Mo_3_C_2_ substrate exhibits no obvious deformation. Overall, the predicted Mo_3_C_2_ slab possesses good thermal stability as an anode for LIBs and SIBs.

## 4. Summary

In summary, in this study, we found a stable 2D non-MXene structure, Mo_3_C_2_, using the USPEX technique. First-principles calculations demonstrated that the Mo_3_C_2_ monolayer not only is thermally, dynamically, and mechanically stable but also exhibits metallic behavior. As an anode material for LIBs and SIBs, the Mo_3_C_2_ possesses the good properties of low diffusion barriers (0.190 eV for Li and 0.118 for Na) and low electrode potentials. The storage capacities are 344 mA h g^−1^ for Li and 306 mA h g^−1^ for Na when adsorbing two layers of adatoms, which are comparable to those of conventional graphite. Moreover, the Mo_3_C_3_ substrate can maintain structural integrity during the AIMD simulations for the Li_18_@(3×3)-Mo_3_C_2_ and Na_18_@(3×3)-Mo_3_C_2_ at 300 and 600 K. All these results support the idea that the 2D Mo_3_C_2_ monolayer can serve as an anode material for LIBs and SIBs.

## Figures and Tables

**Table 1:** The elastic constants *C_ij_* (N/m) and Young’s moduli *Y*^2D^ (N/m) of our predicted Mo_3_C_2_, the Mo_3_C_2_ MXene, graphene [38], BN [38], and SiC [38].

	*C*_11_	*C*_22_	*C*_12_	*C*_66_	$Y_{\left[01\right]}^{2D}$	$Y_{\left[10\right]}^{2D}$
Our predicted Mo_3_C_2_	432.3	432.3	168.4	195.6	366.7	366.7
Mo_3_C_2_ MXene	293.3	293.3	59.7	116.8	281.1	281.1
Graphene	352.7	352.7	60.9	145.9	342.2	342.2
BN	289.8	289.8	63.7	113.1	275.8	275.8
SiC	179.7	179.7	53.9	62.9	163.5	163.5

**Table 2:** Adsorption energies and adatom heights for single Li/Na atom adsorption on the (3×3)-Mo_3_C_2_ monolayer.

	*E*_ad_ (eV)			*h* (Å)		
	C^1^	H	Mo^1^	C^1^	H	Mo^1^
Li@(3×3)-Mo_3_C_2_	−0.467	−0.646	−0.148	1.96	1.66	2.47
Na@(3×3)-Mo_3_C_2_	−0.503	−0.617	−0.453	2.35	2.14	2.76

**Table 3:** Calculated Bader atomic charges in the Mo_3_C_2_ and (Li or Na)@ (3×3)-Mo_3_C_2_ systems.

	Average Charge			
	Mo	C	Li	Na
Mo_3_C_2_	+0.7	−1.1		
Li@(3×3)-Mo_3_C_2_ for C^1^	+0.7	−1.1	+0.9	
Li@(3×3)-Mo_3_C_2_ for H	+0.7	−1.2	+0.9	
Li@(3×3)-Mo_3_C_2_ for Mo^1^	+0.7	−1.1	+0.9	
Na@(3×3)-Mo_3_C_2_ for C^1^	+0.7	−1.1		+0.8
Na@(3×3)-Mo_3_C_2_ for H	+0.7	−1.1		+0.8
Na@(3×3)-Mo_3_C_2_ for Mo^1^	+0.7	−1.1		+0.8

## Data Availability

The original contributions presented in the study are included in the article/Supplementary Materials, further inquiries can be directed to the corresponding authors.

## Supplementary Materials

The following supporting information can be downloaded at: https://www.mdpi.com/article/10.3390/ma17153819/s1, Figure S1. (a) Top and (b) side views of the Mo_3_C_2_ monolayer after 10 ps AIMD simulations at 600 K. Figure S2. Total and partial densities of states of the Mo_3_C_2_ slab. The fermi level is set to zero and marked with the dashed line. Figure S3. Top and side views of the supercells for the most stable configuration of the single-layer 1–18 Li atom(s) adsorbed on the Mo_3_C_2_ surface(s). Figure S4. Top and side views of the supercells for the most stable configuration of the double-layer 19–36 Li atoms adsorbed on the Mo_3_C_2_ surfaces. Figure S5. Top and side views of the supercells for the most stable configuration of the single-layer 1–18 Na atom(s) adsorbed on the Mo_3_C_2_ surface(s). Figure S6. Top and side views of the supercells for the most stable configuration of the double-layer 19–32 Na atoms adsorbed on the Mo_3_C_2_ surfaces.
