# Supplementary material for: Theoretical Investigation of a Novel Two-Dimensional Non-MXene Mo3C2 as a Prospective Anode Material for Li- and Na-Ion Batteries
Source: Materials (Basel). 2024 Aug 2;17(15):3819. doi: 10.3390/ma17153819 (PMC11312968; doi:10.3390/ma17153819)
Supplement: Supplementary file 1 [file materials-17-03819-s001.zip › materials-3097908-supplementary.pdf]

Supplementary Materials

Theoretical Investigation of a Novel Two-Dimensional Non-MXene Mo_3C_2 as a Prospective Anode Material for Li- and Na-Ion Batteries

Bo Xue [1,*], Qingfeng Zeng [2,3,4], Shuyin Yu [2,3] and Kehe Su [5,*]

[1] School of Physical Science and Technology, Northwestern Polytechnical University, Xi'an 710129, China
[2] MSEA International Institute for Materials Genome, Langfang 065500, China; zengqf@dianyunkeji.com (Q.Z.); yusy@dianyunkeji.com (S.Y.)
[3] Particle Cloud Biotechnology (Hangzhou) Co., Ltd., Hangzhou 310018, China
[4] Science and Technology on Thermostructural Composite Materials Laboratory, Northwestern Polytechnical University, Xi'an 710072, China
[5] School of Chemistry and Chemical Engineering, Northwestern Polytechnical University, Xi'an 710129, China
* Correspondence: xuebo@mail.nwpu.edu.cn (B.X.); sukehe@nwpu.edu.cn (K.S.)

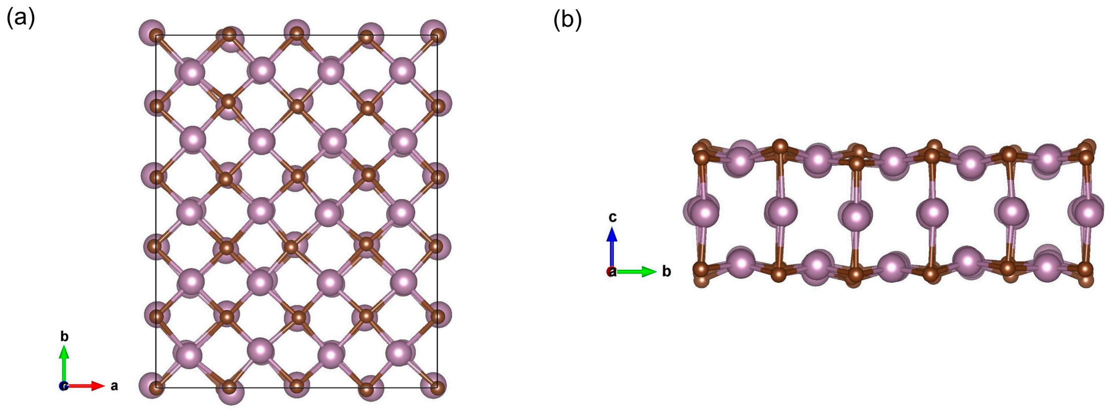

Figure S1. (**a**) Top and (**b**) side views of the Mo_3C_2 monolayer after 10 ps AIMD simulations at 600K.

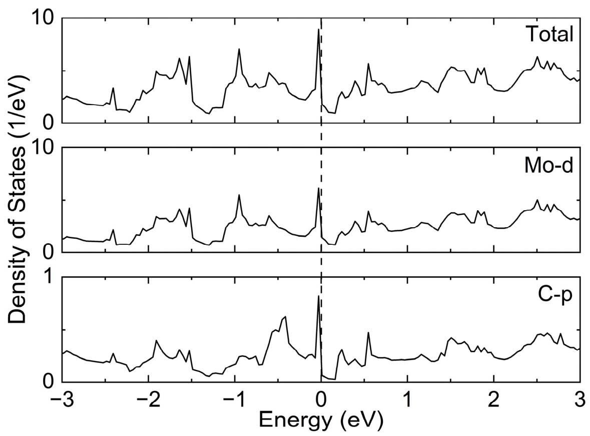

Figure S2. Total and partial densities of states of the Mo_3C_2 slab. The fermi level is set to zero and marked with the dashed line.

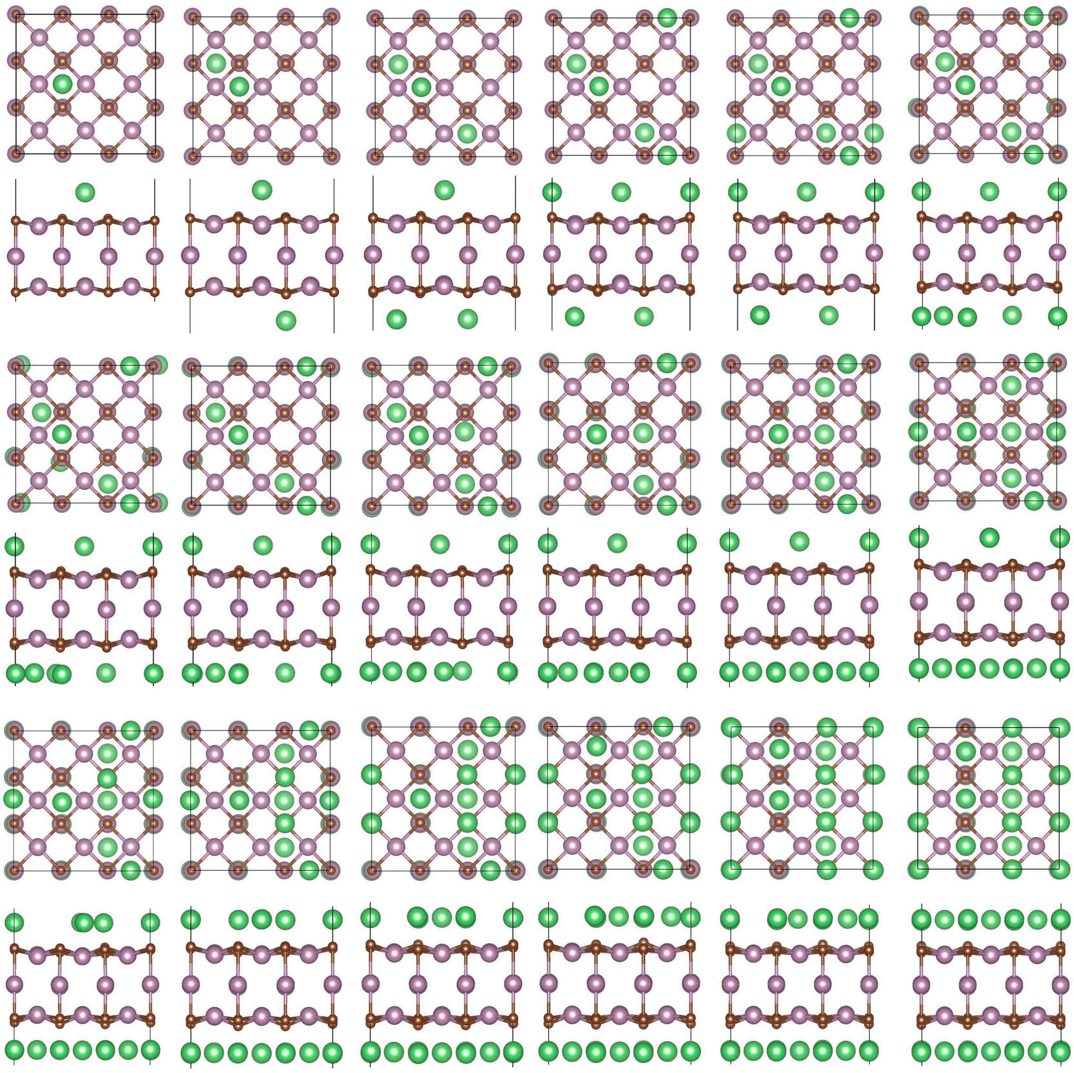

Figure S3. Top and side views of the supercells for the most stable configuration of the single-layer 1-18 Li atom(s) adsorbed on the Mo_3C_2 surface(s).

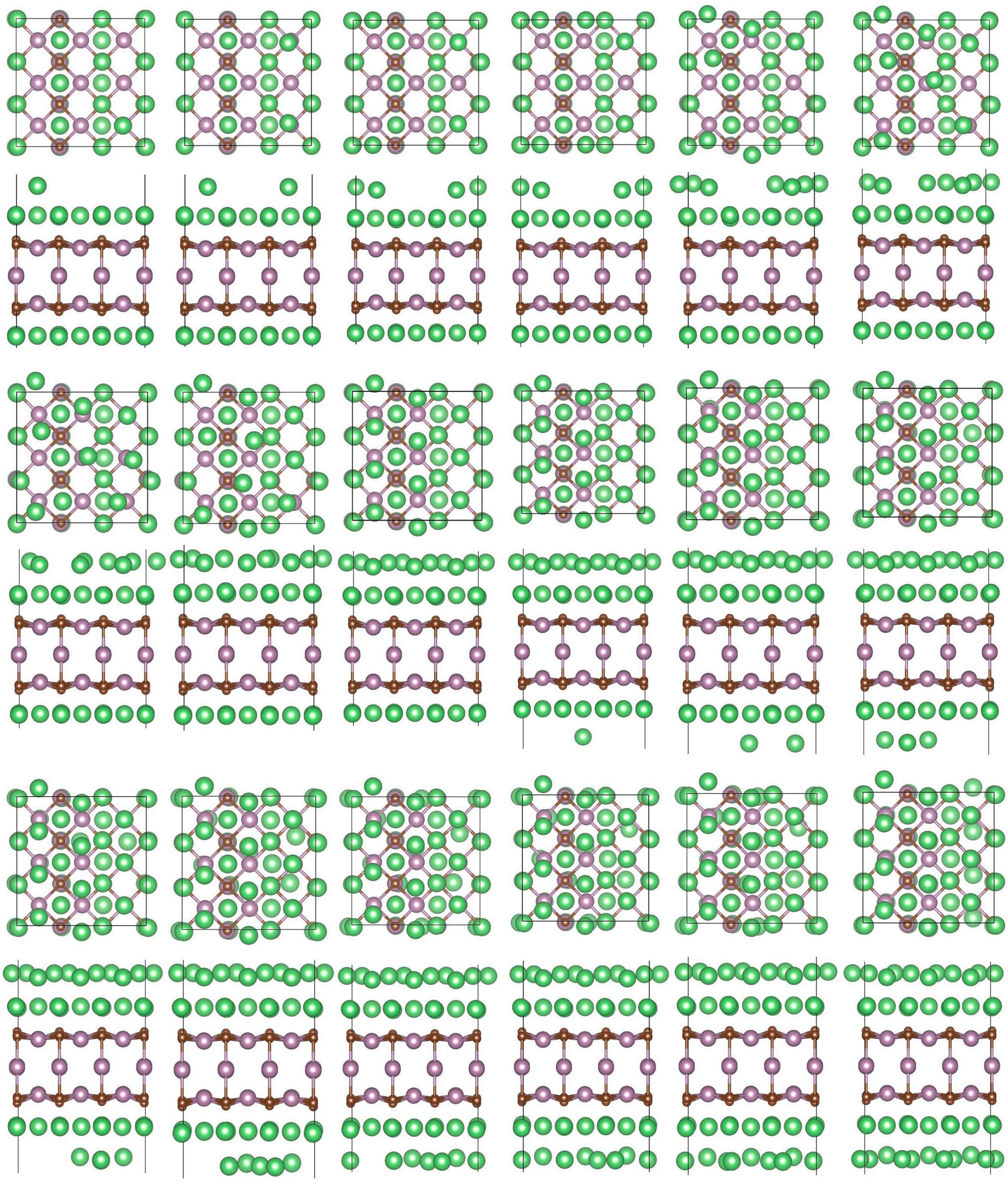

Figure S4. Top and side views of the supercells for the most stable configuration of the double-layer 19-36 Li atoms adsorbed on the Mo_3C_2 surfaces.

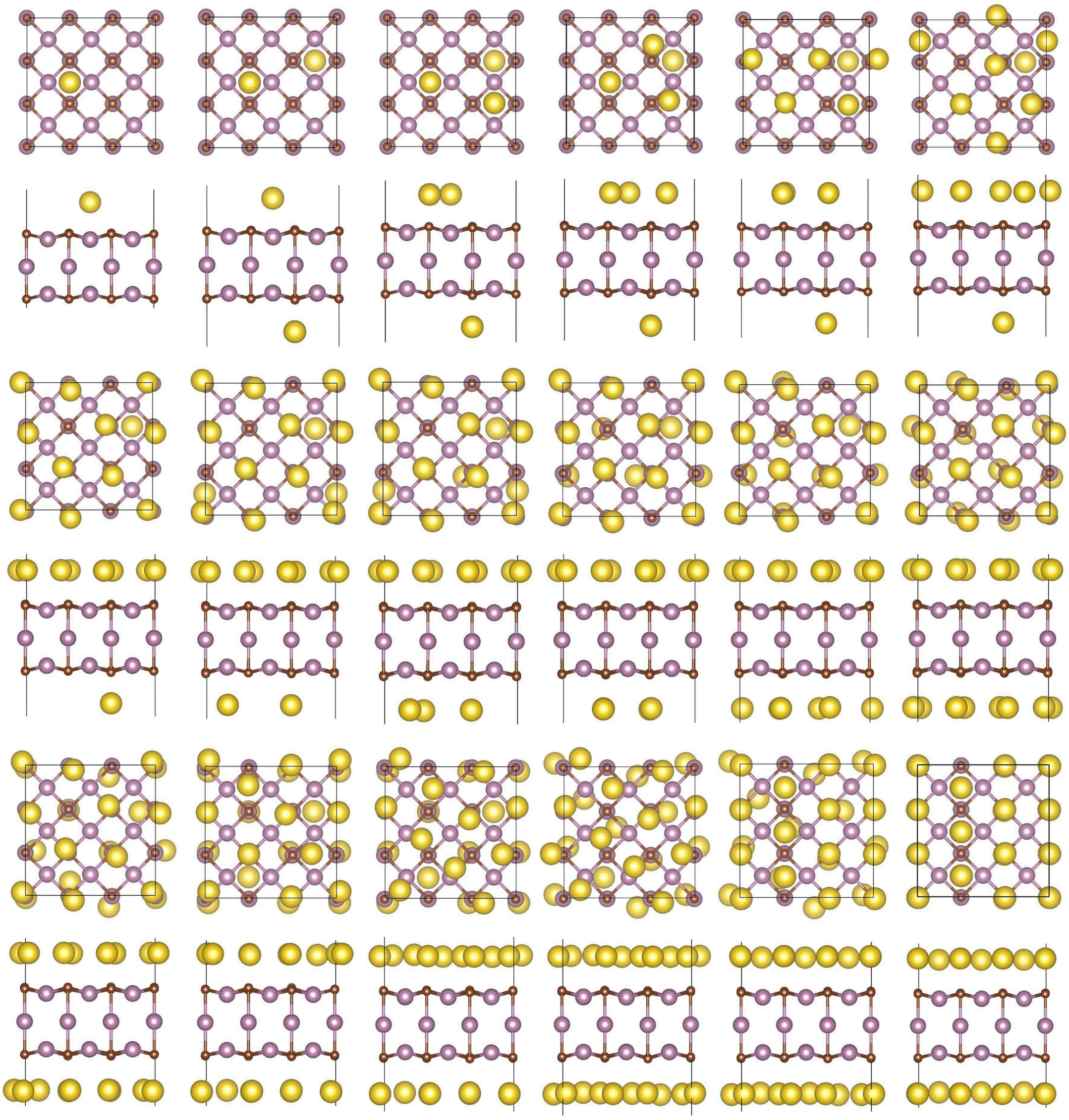

Figure S5. Top and side views of the supercells for the most stable configuration of the single-layer 1-18 Na atom(s) adsorbed on the Mo_3C_2 surface(s).

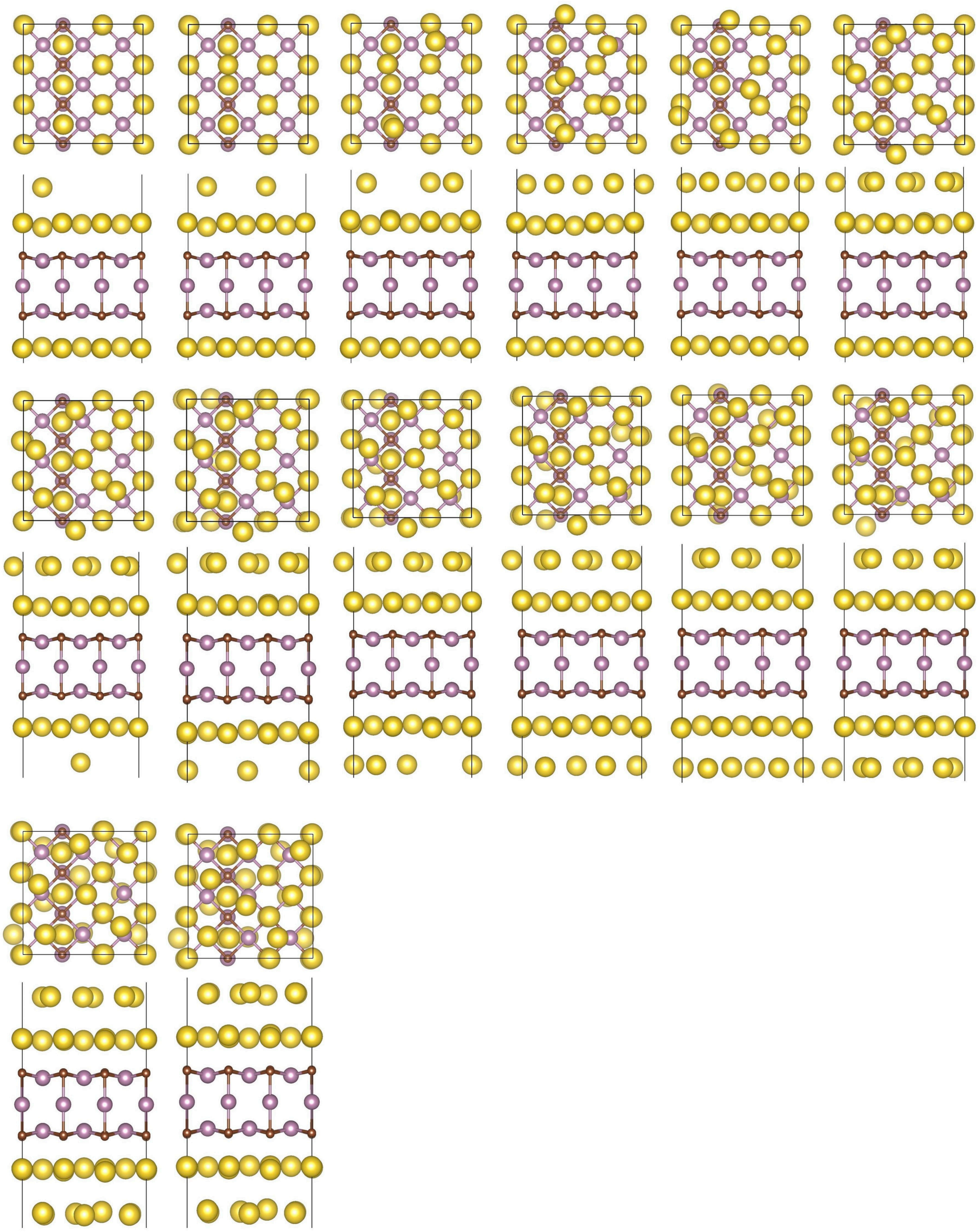

Figure S6. Top and side views of the supercells for the most stable configuration of the double-layer 19-32 Na atoms adsorbed on the Mo_3C_2 surfaces.
